# Does lateral approach preserve the right ventricular function after HeartMate 3 insertion?

**DOI:** 10.1093/icvts/ivad168

**Published:** 2023-10-12

**Authors:** Hideyuki Hayashi, Michael Kirschner, Alice Vinogradsky, Yanling Zhao, Jocelyn Sun, Paul Kurlansky, Melana Yuzefpolskaya, Paolo C Colombo, Gabriel T Sayer, Nir Uriel, Yoshifumi Naka, Koji Takeda

**Affiliations:** Division of Cardiothoracic Surgery, Department of Surgery, Columbia University Medical Center, New York, NY, USA; Division of Cardiothoracic Surgery, Department of Surgery, Columbia University Medical Center, New York, NY, USA; Division of Cardiothoracic Surgery, Department of Surgery, Columbia University Medical Center, New York, NY, USA; Department of Surgery, Center for Innovation and Outcomes Research, Columbia University Medical Center, New York, NY, USA; Department of Surgery, Center for Innovation and Outcomes Research, Columbia University Medical Center, New York, NY, USA; Division of Cardiothoracic Surgery, Department of Surgery, Columbia University Medical Center, New York, NY, USA; Division of Cardiology, Department of Medicine, Columbia University Medical Center, New York, NY, USA; Division of Cardiology, Department of Medicine, Columbia University Medical Center, New York, NY, USA; Division of Cardiology, Department of Medicine, Columbia University Medical Center, New York, NY, USA; Division of Cardiology, Department of Medicine, Columbia University Medical Center, New York, NY, USA; Division of Cardiothoracic Surgery, Department of Surgery, Columbia University Medical Center, New York, NY, USA; Division of Cardiothoracic Surgery, Department of Surgery, Weill Cornell Medical Center, New York, NY, USA; Division of Cardiothoracic Surgery, Department of Surgery, Columbia University Medical Center, New York, NY, USA

**Keywords:** Lateral thoracotomy, Echocardiography, Right ventricular function, Left ventricular assist device

## Abstract

**OBJECTIVES:**

Lateral thoracotomy (LT) approach may preserve the right ventricular (RV) function after left ventricular assist device (LVAD) implantation. This study evaluated the short- and long-term RV function using echocardiography after LVAD implantation via LT or median sternotomy (sternotomy).

**METHODS:**

The patients who underwent HeartMate 3 implantation were retrospectively reviewed. The RV function was assessed before and 1 month and 1 year after LVAD implantation. The primary and secondary outcomes were all-cause mortality and a composite of death or readmission due to RV failure, respectively.

**RESULTS:**

Of the 195 patients, 55 (28%) underwent LT and 140 (72%) underwent sternotomy. There were no significant differences in the preoperative RV geometry or function. One month after the LVAD implantation, the LT group had a smaller RV end-diastolic dimension [42 (29–48) vs 47 (42–52) mm; *P* = 0.003] and RV end-diastolic area [25 (21–28) vs 29 (24–36) cm^2^; *P* < 0.001] and a greater RV fractional area change [30 (25–34)% vs 28 (23–31)%; *P* = 0.04] and peak systolic tissue velocity [8 (7–9) vs 7 (6–8) cm/s; *P* = 0.01]. Twenty-four patients died and 46 met the composite end point. Kaplan–Meier curve analysis did not reveal significant differences between LT and sternotomy in the 2-year survival (93% vs 83%; log-rank test, *P* = 0.28) and adverse event rate (76% vs 71%; log-rank test, *P* = 0.65).

**CONCLUSIONS:**

LT approach yielded a better-preserved RV function at 1 month; however, there were no significant differences in the 2-year survival and adverse event rates.

**Figure:**
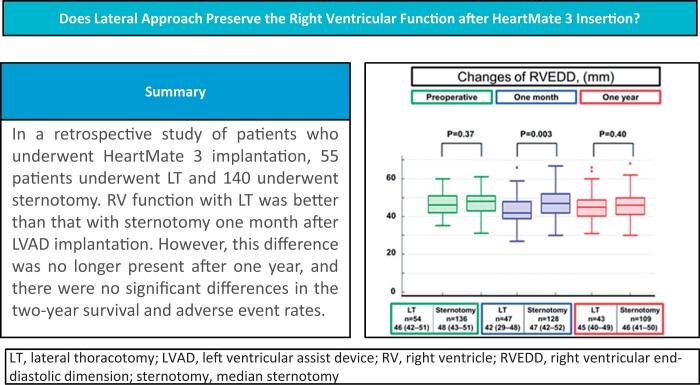

## INTRODUCTION

Minimally invasive cardiac surgery is employed in many cardiac surgical procedures [1, 2]. Recently, advancements in device technology and progressive miniaturization have led to the widespread use of lateral thoracotomy (LT) rather than the traditional median sternotomy (sternotomy) for left ventricular assist device (LVAD) implantation [3, 4].

Previous studies have revealed potential benefits of the LT approach, such as the potentially decreased risk of postoperative right ventricular failure (RVF), presumably due to the preservation of pericardial integrity [1, 2].

To our knowledge, no study has directly evaluated the right ventricular (RV) function and geometry in the patients undergoing HeartMate 3 implantation via either LT or sternotomy.

This study aimed to compare between the 1-month and 1-year RV function and geometry using echocardiogram after HeartMate 3 implantation via LT or sternotomy.

## MATERIALS AND METHODS

### Ethical statement

This retrospective study was approved by the Institutional Review Board of Columbia University Medical Center (AAAU 2877, October 2022), and the requirement for individual consent was waived.

### Patients and data collection

The medical records of consecutive patients who underwent HeartMate 3 implantation (Abbott Inc., Chicago, IL, USA) at Columbia University Medical Center between November 2014 and September 2021 were retrospectively reviewed. The exclusion criteria were the patients who: (1) received right ventricular assist devices (RVADs) intraoperatively; (ii) had a history of tricuspid valve (TV) surgery; or (iii) had undergone concomitant TV surgery at the time of LVAD implantation. Clinical, electrocardiographic, laboratory and haemodynamic data were collected from electronic medical records.

The primary outcome was all-cause mortality. The secondary outcome was a composite of death or readmission due to worsening RVF within 2 years of implantation. RVF was defined according to the Interagency Registry for Mechanically Assisted Circulatory Support based on the signs and symptoms of RVF and elevated central venous pressure [5]. If a patient experienced more than one of these events, the time to the first event was used as the composite outcome.

### Transthoracic echocardiogram examination and right ventricular geometry and function

All transthoracic echocardiograms (TTEs) were performed by a trained, registered cardiac sonographer before LVAD implantation and 1 month and 1 year after LVAD implantation. TTEs were interpreted by experienced attending physicians at our institution according to the American Society of Echocardiography guidelines under a rigid quality-control regimen. Device speed optimization studies were routinely performed at least 2 weeks after LVAD implantation and before hospital discharge. The device speed was determined based on the TTE findings [6]. The optimal pump speed was defined as the highest speed that allowed the neutral alignment of the interventricular septum and intermittent aortic valve opening without increased mitral regurgitation. If a patient underwent several TTEs during the study period, only the data from the TTE closest to the LVAD implantation, 1 month and 1 year after device placement were analysed. One-year follow-up TTEs were performed at least 6 months after LVAD implantation.

**Figure 1:**
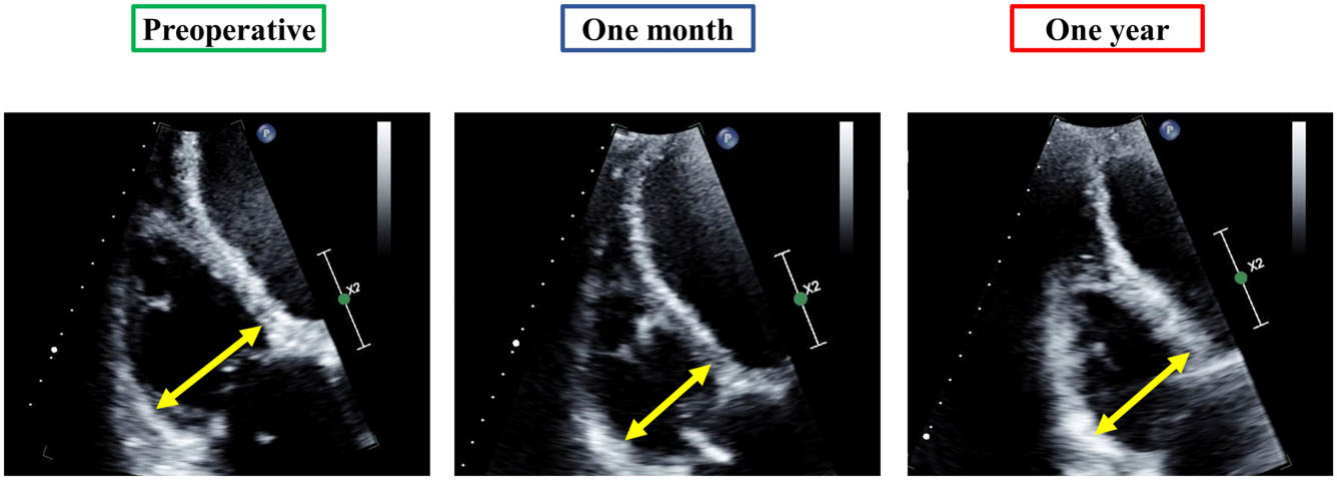
Changes in the RV geometry after LT approach in modified apical four-chamber views. RVEDD: preoperative, 50 mm; 1 month, 42 mm; 1 year, 47 mm. RVEDA: preoperative, 40 cm^2^; 1 month, 28 cm^2^; 1 year, 32 cm^2^. Yellow arrow: RVEDD. LT: lateral thoracotomy; RV: right ventricle; RVEDA: right ventricular end-diastolic area; RVEDD: right ventricular end-diastolic dimension.

RV geometry was assessed via the RV end-diastolic dimension (RVEDD) and RV end-diastolic area (RVEDA) using modified apical four-chamber views encompassing the entire RV [7] (Fig. 1). RV function was evaluated quantitatively using the RV fractional area change and peak systolic tissue velocity of the RV lateral wall. The severity of tricuspid regurgitation (TR) and mitral regurgitation was divided into 4 grades (none to trivial, mild, moderate and severe) based on recommendations from the American Society of Echocardiography [8]. Left ventricular (LV) ejection fraction, LV end-diastolic dimension, LV end-systolic dimension, LV mass index and left atrial volume index were measured in accordance with the previously published guidelines [9]. All variables were acquired with at least 3 beats and averaged.

### Surgical management

Between 2014 and 2019, full sternotomy was the standard approach for HeartMate 3 implantation. Since 2020, LT has become the standard approach for HeartMate 3 implantation. LT was performed via an upper J-hemisternotomy in the third right intercostal space or right anterior thoracotomy in the second or third intercostal space with left thoracotomy in the fifth or sixth intercostal space. Computed tomography scans were routinely obtained before surgery to evaluate the access to the ascending aorta and LV apex [10]. During this time, sternotomies continued to be performed in the patients undergoing concomitant procedures (except for aortic valve repair), such as TV surgery or simultaneous RVAD implantation. Concomitant TV surgery is generally performed for patients with severe TR, although this decision is left to the discretion of the surgeon. All cases were performed with cardiopulmonary bypass regardless of the approach.

### Statistical analysis

Normality of all continuous variables was examined using the Shapiro–Wilk test. Continuous variables are presented as means with standard deviations if normally distributed or otherwise as medians with interquartile ranges. Categorical variables are presented as numbers and proportions. Student’s *t*-test (for normally distributed data) or Mann–Whitney test (for non-normally distributed data) was used to compare the continuous variables between the LT and sternotomy groups. Paired *t*-test or Wilcoxon signed-rank test was used to compare the parameters of the preoperative and 1-month and 1-year follow-up TTE, as appropriate. Chi-squared test or Fisher’s exact test was used to compare the difference in dichotomous variables depending on the size (>5). The Kaplan–Meier method was used to estimate the overall survival and event-free survival, and groups were compared using the log-rank test.

Propensity score matching was performed with the surgical approach as the dependent variable and selected variables from Table 1 as independent variables in the model. Patients were matched in a 1:1 ratio using nearest-neighbor methods with no replacement and with a calliper of 0.25 width. Matching balance was determined via a standardized mean difference of <0.2 [11].

**Table 1:** Patients’ characteristics

Variables	LT, *n* = 55	Sternotomy, *n* = 140	*P*-value
Age (years)	61 (55–70)	61 (53–69)	0.80
Female sex (%)	9 (16%)	22 (16%)	0.91
White (%)	20 (36%)	83 (59%)	0.004
BSA (m^2^)	2.1 (1.9–2.2)	2.0 (1.8–2.2)	0.95
Hypertension (%)	41 (75%)	92 (66%)	0.24
Diabetes mellitus (%)	28 (51%)	55 (39%)	0.14
Dyslipidaemia (%)	32 (58%)	90 (64%)	0.43
COPD (%)	3 (6%)	17 (12%)	0.17
Atrial fibrillation (%)	23 (42%)	71 (51%)	0.27
ICD (%)	41 (75%)	112 (80%)	0.41
Ischaemic cardiomyopathy	27 (49%)	59 (42%)	0.38
Intention to treat			0.16
Bridge to transplant (%)	6 (11%)	27 (19%)	
Destination therapy (%)	49 (89%)	113 (81%)	
INTERMACS profile level 1 or 2 (%)	28 (51%)	83 (59%)	0.29
Laboratory parameters			
BUN (mg/dl)	26.0 (20.3–33.8)	22.5 (17.0–33.0)	0.07
Creatinine (mg/dl)	1.3 (1.1–1.6)	1.4 (1.1–1.7)	0.74
Albumin (g/dl)	3.6 ± 0.5	3.8 ± 0.5	0.01
Total bilirubin (mg/dl)	0.8 (0.5–1.1)	0.7 (0.5–1.0)	0.84
Haemoglobin (g/dl)	11.2 ± 2.3	11.8 ± 2.3	0.14
Haemodynamic parameters			
Mean PA pressure (mmHg)	32.7 ± 11.9	34.7 ± 10.3	0.24
PCWP (mmHg)	21.3 ± 8.7	22.8 ± 8.7	0.29
RA pressure (mmHg)	9.8 ± 7.3	9.6 ± 5.9	0.87
Cardiac index (l/min/m^2^)	2.1 (1.7–2.4)	1.8 (1.5–2.2)	0.004
PVR (wood units)	2.7 (1.5–3.6)	3.3 (2.1–4.6)	0.02
RV stroke work index (g·m/m^2^/beat)	7.4 (5.3–9.7)	7.6 (5.6–10.1)	0.38
PA pulsatility index	3.4 (2.1–4.7)	3.4 (2.1–5.3)	0.79
Concomitant surgery			
Coronary artery bypass grafting (%)	0 (0%)	2 (1%)	0.37
Aortic valve repair (%)	8 (15%)	29 (21%)	0.32
Mitral valve repair (%)	0 (0%)	21 (15%)	0.002
ASD or PFO closure (%)	0 (0%)	11 (8%)	0.03
Ablation (%)	0 (0%)	5 (4%)	0.16
Left atrial appendage ligation (%)	0 (0%)	81 (58%)	<0.001

Values are expressed as mean ± standard deviation, median (interquartile range) or numbers (%).

ASD: atrial septal defect; BSA: body surface area; BUN: blood urea nitrogen; COPD: chronic obstructive pulmonary disease; ICD: implantable cardioverter defibrillator; INTERMACS: Interagency Registry for Mechanically Assisted Circulatory Support; LT: lateral thoracotomy; PA: pulmonary artery; PCWP: pulmonary capillary wedge pressure; PFO: patent foramen ovale; PVR: pulmonary vascular resistance; RA: right atrium; RV: right ventricle; sternotomy: median sternotomy.

All statistical analyses were performed using MedCalc (version 15.8; MedCalc Software, Ostend, Belgium) and R statistical software (version 4.1.1; R Foundation, Vienna, Austria).

## RESULTS

### Baseline characteristics

**Figure 2:**
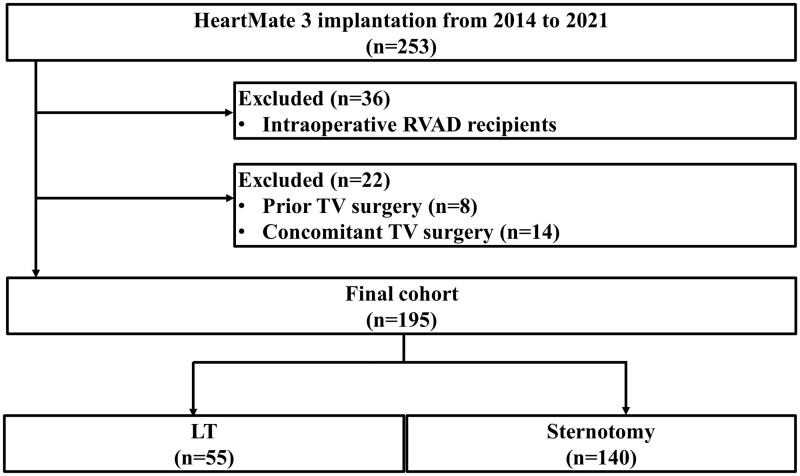
Flow diagram of the selection process of the patients. LT: lateral thoracotomy; LVAD: left ventricular assist device; RVAD: right ventricular assist device; sternotomy: median sternotomy; TV: tricuspid valve.

A total of 253 patients underwent HeartMate 3 implantation during the 7-year study period. A flowchart of the study population is shown in Fig. 2. The 36 patients who underwent RVAD intraoperatively and the 22 patients who had undergone concomitant TV surgery or had a history of TV surgery were excluded from the analysis. Of the 195 patients included in the analysis, 55 (28%) underwent LT and 140 (72%) underwent sternotomy.

While the LT group was less likely to be from a White origin compared to the sternotomy group [20 (36%) vs 83 (59%); *P* = 0.004], there were no significant baseline differences in age, sex, body size, Interagency Registry for Mechanically Assisted Circulatory Support profile level or treatment strategy between the 2 groups (Table 1). Regarding the preoperative haemodynamic parameters, the LT group had a greater cardiac index compared to that of the sternotomy group [2.1 (1.7–2.4) vs 1.8 (1.5–2.2) l/min/m^2^]. There were no significant differences in the pulmonary artery pressure, pulmonary capillary wedge pressure, pulmonary artery pulsatility index, RV stroke work index or right atrial pressure between the 2 groups.

### Echocardiographic parameters

**Figure 3:**
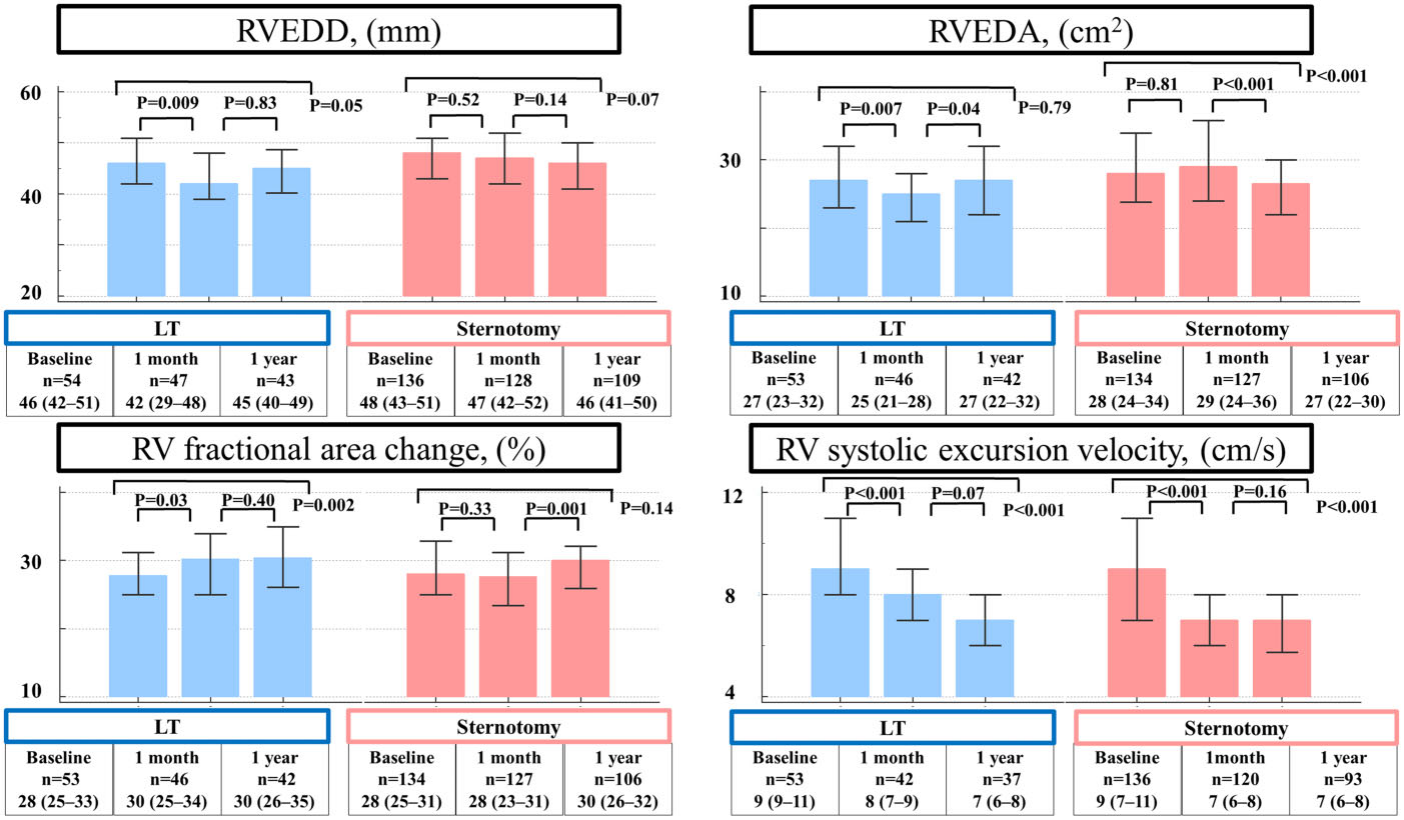
Changes in the RV geometry. Preoperative, 1 month and 1 year before the LVAD implantation and 1 month and 1 year after the LVAD implantation. LT: lateral thoracotomy; LVAD: left ventricular assist device; RV: right ventricle; RVEDA: right ventricular end-diastolic area; RVEDD: right ventricular end-diastolic dimension; sternotomy: median sternotomy.

Preoperative TTEs were performed at a median of 7 (3–15) days preoperatively, whereas short-term and long-term follow-up TTEs were performed at 35 (27–47) and 360 (304–392) days postoperatively, respectively. The TTE findings are summarized in Table 2, Supplementary Material, Table S1 and Fig. 3.

**Table 2:** Changes in the tricuspid regurgitation and right ventricle parameters

	LT	Sternotomy	*P*-value
Preoperative parameters			
RVEDD (mm), *n* = 54 vs 136	46 (42–51)	48 (43–51)	0.37
RVEDA (cm^2^), *n* = 53 vs 134	27 (23–32)	28 (24–34)	0.29
RVFAC (%), *n* = 53 vs 134	28 (25–33)	28 (25–31)	0.46
RV systolic excursion velocity (cm/s), *n* = 53 vs 136	9 (9–11)	9 (7–11)	0.63
TR severity (%), *n* = 55 vs 140			0.16
None to trivial	16 (29%)	51 (36%)	
Mild	19 (35%)	59 (42%)	
Moderate	15 (27%)	25 (18%)	
Severe	5 (9%)	5 (4%)	
TR moderate or severe (%)	20 (36%)	30 (22%)	0.03
One-month postoperative parameters			
RVEDD (mm), *n* = 47 vs 128	42 (29–48)	47 (42–52)	0.003
RVEDA (cm^2^), *n* = 46 vs 127	25 (21–28)	29 (24–36)	<0.001
RVFAC (%), *n* = 46 vs 127	30 (25–34)	28 (23–31)	0.04
RV systolic excursion velocity (cm/s), *n* = 42 vs 120	8 (7–9)	7 (6–8)	0.01
TR severity (%), *n* = 52 vs 135			0.05
None to trivial	20 (38%)	65 (48%)	
Mild	19 (37%)	49 (36%)	
Moderate	6 (12%)	17 (13%)	
Severe	7 (13%)	4 (3%)	
TR moderate or severe (%)	13 (25%)	21 (16%)	0.14
One-year postoperative parameters			
RVEDD (mm), *n* = 43 vs 109	45 (40–49)	46 (41–50)	0.40
RVEDA (cm^2^), *n* = 42 vs 106	27 (22–32)	27 (22–30)	0.42
RVFAC (%), *n* = 42 vs 106	30 (26–35)	30 (26–32)	0.39
RV systolic excursion velocity (cm/s), *n* = 37 vs 93	7 (6–8)	7 (6–8)	0.09
TR severity (%), *n* = 46 vs 113			0.87
None to trivial	32 (70%)	78 (69%)	
Mild	7 (15%)	22 (20%)	
Moderate	3 (7%)	5 (4%)	
Severe	4 (8%)	8 (7%)	
TR moderate or severe (%)	7 (15%)	13 (11%)	0.54

Values are expressed as medians (interquartile range) or numbers (%).

LT: lateral thoracotomy; RV: right ventricle; RVEDA: right ventricular end-diastolic dimension; RVEDD: right ventricular end-diastolic dimension; RVFAC, right ventricular fractional area change; sternotomy: median sternotomy; TR: tricuspid regurgitation.

There were no significant differences in the preoperative RV geometry [RVEDD, 46 (42–51) vs 48 (43–51) mm; *P* = 0.37 and RVEDA, 27 (23–32) vs 28 (24–34) cm^2^; *P* = 0.29], RV function [RV fractional area change, 28 (25–33)% vs 28 (25–31)%; *P* = 0.46] and peak systolic tissue velocity [9 (9–11) vs 9 (7–11) cm/s; *P* = 0.63] between the LT and the sternotomy groups.

One month after the LVAD implantation, the LT group had a smaller RVEDD [42 (29–48) vs 47 (42–52) mm; *P* = 0.003] and RVEDA [25 (21–28) vs 29 (24–36) cm^2^; *P* < 0.001] and greater RV fractional area change [30 (25–34)% vs 28 (23–31)%; *P* = 0.04] and peak systolic tissue velocity [8 (7–9) vs 7 (6–8) cm/s; *P* = 0.01]. Moreover, 1 month after the LVAD implantation, the LVAD speed was similar between the 2 groups [5500 (5313–5700) vs 5450 (5300–5600) rpm; *P* = 0.63].

One year after the LVAD placement, there were no significant differences in the RV geometry [RVEDD, 45 (40–49) vs 46 (41–50); *P* = 0.40 and RVEDA, 27 (22–32) vs 27 (22–30) cm^2^; *P* = 0.42], RV function [RV fractional area change, 30 (26–35)% vs 30 (26–32)%; *P* = 0.39] or peak systolic tissue velocity [7 (6–8) vs 7 (6–8) cm/s; *P* = 0.09] between the 2 groups.

In the LT group, the RVEDDs and RV fractional area changes in the 1-month postoperative TTE were smaller and higher compared to those of the preoperative TTE. These differences were not observed in the sternotomy group. Postoperative RV peak systolic tissue velocities at 1 month and 1 year postoperatively TTE were lower than those of the preoperative values in both groups.

When compared with the patients in the sternotomy group, the patients in the LT group had a significantly higher prevalence of moderate or severe TR [20 (36%) vs 30 (22%); *P* = 0.03] at preoperative TTE (Table 2 and Supplementary Material, Fig. S1).

### Clinical outcomes

In our cohort, 24 patients died and 46 met the composite end point with a 100% follow-up rate at 2 years. Twenty-five patients were hospitalized during the study period due to RVF. The causes of death were sepsis and/or multiorgan dysfunction [11 patients (LT: *n* = 1, sternotomy: *n* = 10)], quality of life concerns [3 patients (LT: *n* = 1, sternotomy: *n* = 2)], device failure [3 patients (LT: *n* = 1, sternotomy: *n* = 2)], stroke [3 patients (LT: *n* = 0, sternotomy: *n* = 3)], withdrawal of care due to advanced cancer [2 patients (LT: *n* = 0, sternotomy: *n* = 2)], bleeding [1 patient (LT: *n* = 1, sternotomy: *n* = 0)] and unknown [1 patient (LT: *n* = 0, sternotomy: *n* = 1)].

**Figure 4:**
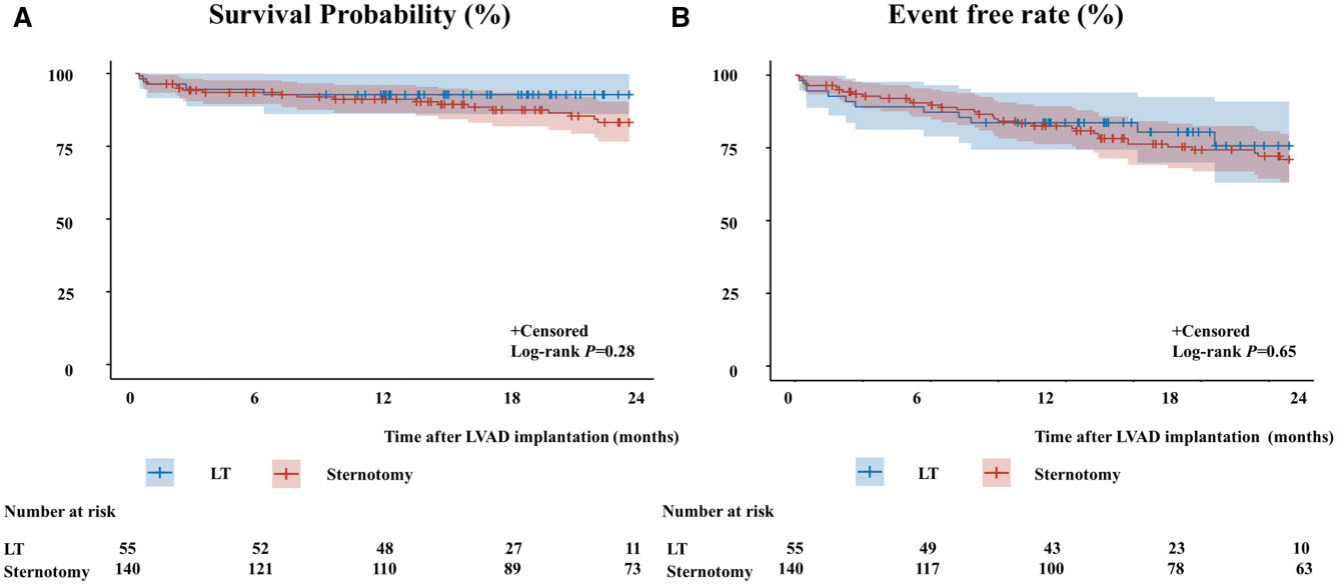
Kaplan–Meier analysis of mortality (**A**) and composite outcome (**B**). LT: lateral thoracotomy; LVAD: left ventricular assist device; sternotomy: median sternotomy.

Kaplan–Meier curve analysis demonstrated a 2‐year survival of 93% and 83% in the LT and sternotomy groups, respectively (log-rank test, *P* = 0.28) (Fig. 4). Curve analysis showed 2‐year event-free rates of 76% and 71% in the LT and sternotomy groups, respectively (log-rank test, *P* = 0.65).

### Propensity score matching for patients with lateral thoracotomy and sternotomy

After matching the patients’ baseline characteristics and operative details, there were 36 patients in each group (Supplementary Material, Table S2). Short-term better RV function and geometry were also observed in the matching cohort (Supplementary Material, Table S3). Kaplan–Meier curve showed no difference in the 2-year survival and composite events between the 2 groups (Supplementary Material, Fig. S2). However, the patients with sternotomy required more transfusions (Supplementary Material, Table S4).

## DISCUSSION

The LT Group preserved better RV function and geometry 1 month after LVAD implantation than those of the sternotomy group. However, these differences were not observed at the 1-year follow-up, and there were no significant differences in the 2-year survival and RVF rates.

Our findings suggest that LT may confer a short-term RV protective strategy [1, 2]. Potential explanations for this phenomenon include preservation of the pericardium, which allows for the maintenance of RV geometry and existing pressure–volume relationships and decreased direct cardiac manipulation intraoperatively [1–3]. Pericardial integrity may be crucial, as the avascular fibrous sac surrounding the LV and RV plays a significant role in maintaining the cardiac position and supporting the LV twist and longitudinal septal motion [12–14]. When pericardial incisions are made, the RV longitudinal function and peak systolic tissue velocity of the RV lateral wall decrease, although longitudinal shortening accounts for a greater proportion of RV shortening than that of LV shortening [15, 16]. Moreover, the RV function after the LVAD implantation is more dependent on the transverse motion of the RV free wall than on the longitudinal motion [17]. In the present study, the postoperative peak systolic tissue velocities decreased in both groups when compared with their respective preoperative values. Although changes secondary to pericardial incision may persist for a certain duration, no significant differences were observed in the RV function and geometry 1 year postoperatively [18].

Therefore, the mechanism underlying the differences in short-term RV function likely involves excess RV preload and significant TR.

Haemodynamically, the LVAD therapy causes dramatic changes as the cardiac output is restored and LV filling pressures are relieved. This can in turn lead to decreased RV afterload and increased RV preload [19, 20]. Compared with the LV, the RV originally exhibits higher sensitivity to afterload changes because it pumps against a lower pressure of pulmonary circulation with a lower ventricular mass and a distinct myocardial metabolism [21, 22]. Additionally, alterations in RV geometry following LVAD insertion can cause RV dysfunction, and changes in interventricular septal motion, secondary to decreased LV pressure, accelerate bowing of the septum towards the LV [23]. RV contractility partially relies on LV contraction as the 2 ventricles share a common wall. LV dysfunction may also cause increases in RV afterload and size, ultimately leading to RVF [23]. Accordingly, the RV function after LVAD implantation may become more sensitive to the RV preloading.

A recent expert consensus declared that patients requiring concomitant TV surgery should not be considered for LT [24]. In our study, the 14 patients who underwent concomitant TV surgery via sternotomy were excluded as the procedure could affect the RV function and morphology. Consequently, the LT group had a significantly greater prevalence of moderate or severe TR preoperatively and severe TR 1 month postoperatively than that of the sternotomy group. In these patients, TR could contribute to volume overload, leading to further RV dysfunction and dilatation, and acceleration of a vicious cycle that negatively impacts prognosis [16, 25]. In the present study, patients with preoperative significant TR had worse outcomes (Supplementary Material, Fig. S3). There were no significant differences in the 2-year survival, adverse event rates and long-term RV function despite the better RV function and geometry at 1 month in the LT group. Nevertheless, the preoperative and residual TR after 1 month may negatively influence the long-term function, leading to subsequent RVF and death.

Short-term better RV function and geometry were also observed in the matching cohort, which had no significant differences in the baseline TR grade and concomitant surgery. Consistent with the previous study, LT decreased the RV preload and blood product requirements compared with sternotomy in the matching cohort [1].

Ultimately, our study may suggest that the LT approach should be considered for selected patients undergoing LVAD placement to take advantage of its potential short-term beneficial effects with respect to the RV function and geometry. Long-term surveillance and monitoring will help determine the impact of significant TR on the clinical outcomes in this cohort.

### Limitations

This study has some limitations. The design was retrospective, single centre and observational with a small sample size, particularly for the LT group and events. Echocardiographic RV assessment may be hindered by anatomical complexity, presence of devices and severe manifestations of end-stage heart failure [17]. Additionally, we could not evaluate all the parameters of interest in the patients with poor imaging quality, which prevented proper assessment of function and geometry, even after excluding those with a history of TV surgery or concomitant TV surgery.

## CONCLUSION

Our preliminary data suggest that after 1 month from LVAD implantation, the RV function was better preserved in patients who underwent LT than in those who underwent sternotomy. At the 1-year postoperative evaluation, this difference was no longer present, and there were no significant differences in the 2-year survival and adverse event rates.

## Supplementary Material

ivad168_Supplementary_Data

## Data Availability

The data will be shared on reasonable request to the corresponding author.

## ABBREVIATIONS

LV: Left ventricular
LVAD: Left ventricular assist device
LT: Lateral thoracotomy
RV: Right ventricular
RVADs: Right ventricular assist devices
RVF: Right ventricular failure
RVEDA: Right ventricular end-diastolic area
RVEDD: Right ventricular end-diastolic dimension
sternotomy: Median sternotomy
TR: Tricuspid regurgitation
TTEs: Transthoracic echocardiograms
TV: Tricuspid valve
