# Supplementary figures and images for: Does lateral approach preserve the right ventricular function after HeartMate 3 insertion?
Source: Interdiscip Cardiovasc Thorac Surg. 2023 Oct 12;37(4):ivad168. doi: 10.1093/icvts/ivad168 (PMC10612129; doi:10.1093/icvts/ivad168)

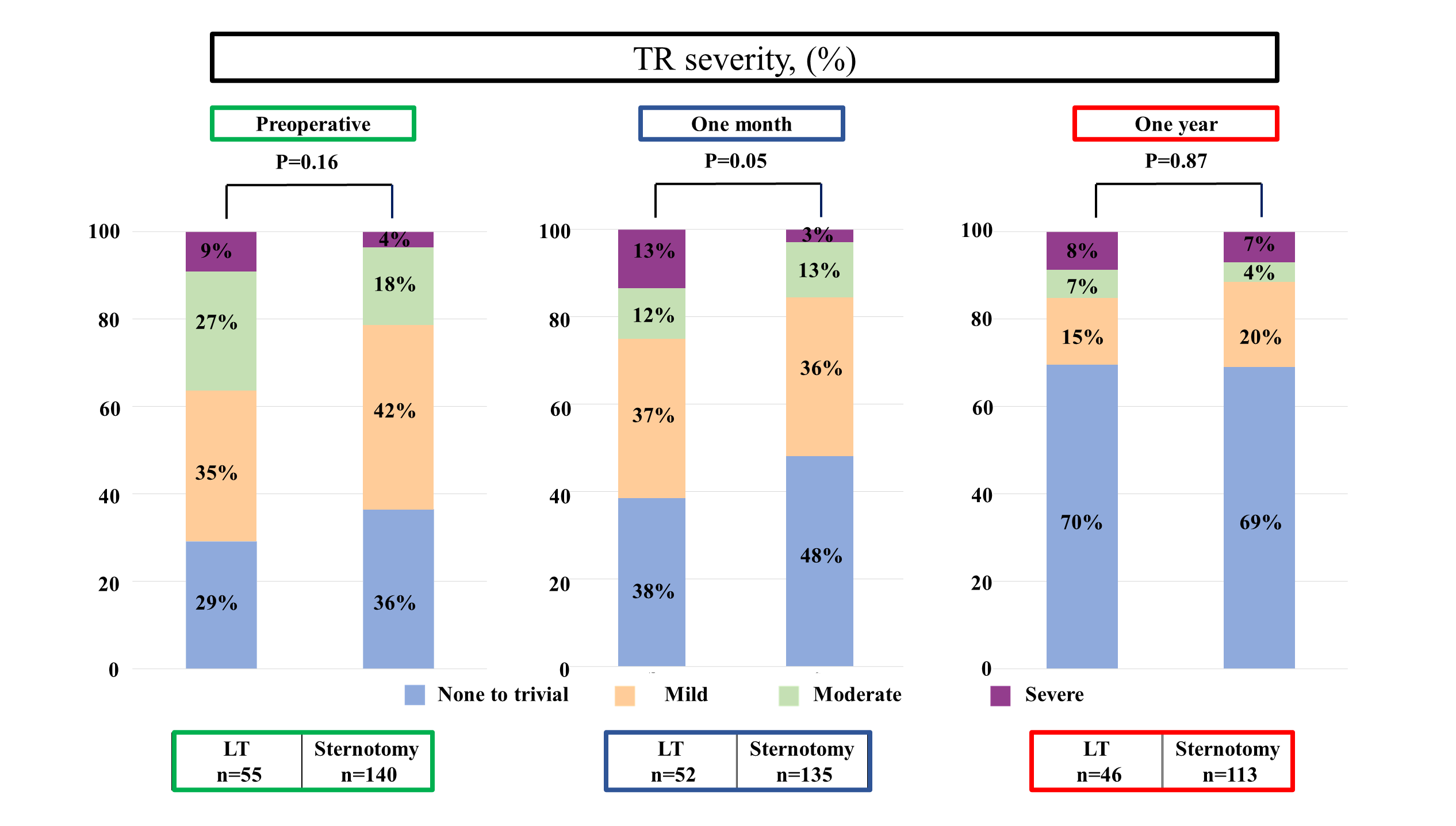

Supplement: ivad168_Supplementary_Data [file ivad168_supplementary_data.zip › Figure S1.tif]
